# Intragraft B cell differentiation during the development of tolerance to kidney allografts is associated with a regulatory B cell signature revealed by single cell transcriptomics

**DOI:** 10.1016/j.ajt.2023.05.036

**Published:** 2023-06-08

**Authors:** Michael Tyler Guinn, Edward S. Szuter, Takahiro Yokose, Jifu Ge, Ivy A. Rosales, Kashish Chetal, Ruslan I. Sadreyev, Alex G. Cuenca, Daniel Kreisel, Peter T. Sage, Paul S. Russell, Joren C. Madsen, Robert B. Colvin, Alessandro Alessandrini

**Affiliations:** 1 Michael E. DeBakey Department of Surgery, Baylor College of Medicine, Houston, Texas, USA; 2 Center for Transplantation Sciences, Department of Surgery, Massachusetts General Hospital, Boston, Massachusetts, USA; 3 Boston’s Children Hospital, Harvard Medical School, Boston, Massachusetts, USA; 4 Department of Pathology, Massachusetts General Hospital, Boston, Massachusetts, USA; 5 Department of Molecular Biology, Massachusetts General Hospital, Boston, Massachusetts, USA; 6 Departments of Surgery, Pathology, and Immunology, Washington University School of Medicine, St. Louis, Missouri, USA; 7 Transplantation Research Center, Renal Division, Brigham and Women’s Hospital, Harvard Medical School, Boston, Massachusetts, USA; 8 Division of Cardiac Surgery, Massachusetts General Hospital, Boston, Massachusetts, USA

**Keywords:** tolerance, B cell biology, immunogenetics, informatics, kidney transplantation, molecular biology

## Abstract

Mouse kidney allografts are spontaneously accepted in select, fully mismatched donor-recipient strain combinations, like DBA/2J to C57BL/6 (B6), by natural tolerance. We previously showed accepted renal grafts form aggregates containing various immune cells within 2 weeks posttransplant, referred to as regulatory T cell–rich organized lymphoid structures, which are a novel regulatory tertiary lymphoid organ. To characterize the cells within T cell–rich organized lymphoid structures, we performed single-cell RNA sequencing on CD45^+^ sorted cells from accepted and rejected renal grafts from 1-week to 6-months posttransplant. Analysis of single-cell RNA sequencing data revealed a shifting from a T cell–dominant to a B cell–rich population by 6 months with an increased regulatory B cell signature. Furthermore, B cells were a greater proportion of the early infiltrating cells in accepted vs rejecting grafts. Flow cytometry of B cells at 20 weeks posttransplant revealed T cell, immunoglobulin domain and mucin domain-1^+^ B cells, potentially implicating a regulatory role in the maintenance of allograft tolerance. Lastly, B cell trajectory analysis revealed intragraft differentiation from precursor B cells to memory B cells in accepted allografts. In summary, we show a shifting T cell– to B cell–rich environment and a differential cellular pattern among accepted vs rejecting kidney allografts, possibly implicating B cells in the maintenance of kidney allograft acceptance.

## 1. Introduction

One of the major aims in the field of transplantation is to identify reliable methods to induce allograft tolerance in recipient hosts.^1–3^ When this goal was achieved, negative consequences such as chronic rejection and immunosuppressive side effects could be avoided altogether.^4,5^ To obtain control over tolerance development, however, requires understanding the mechanism by which it develops naturally if recapitulating such a biological process is to be successful. While short-term outcomes have improved in kidney transplantation, alloimmune responses remain responsible for late failure of many grafts in addition to immunosuppressive drug toxicity. Approximately 3% of kidney transplants are lost per year despite ongoing maintenance of immunosuppression.^6^ This loss has been traditionally thought to be primarily generated in secondary lymphoid organs^7,8^; however, it has been increasingly recognized that both deleterious and beneficial alloimmune responses can be regulated locally at the level of transplanted grafts.^9^ Recent studies have analyzed the development of tolerance in various allografts, including kidneys, livers, and hearts.^1,10–12^ One such model includes murine strain combinations Dilute Brown Agouti mouse strain 2j (DBA/2J) and mouse number 57 strain (C57BL/6), in which kidneys are spontaneously accepted.^13–15^

In previous work, we used this model to show that accepted kidney allografts develop periarterial aggregates of lymphocytes, which represent novel regulatory tertiary lymphoid organs (rTLOs), similar to tertiary lymphoid structures found in solid tumors.^10–13^ Bulk RNA gene expression analysis of these rTLOs previously revealed a Foxp3^+^ RNA and protein signature in regulatory T cells (Treg), which has been labeled Treg-rich organized lymphoid structures (TOLS).^10^ Interestingly, when Tregs are depleted in transplant recipients in the first few months after transplantation, TOLS dissolve and acute T cell-mediated rejection is precipitated.^14^

In addition to Tregs, regulatory B cells (Bregs) can provide similar immunosuppressive properties and promote a pro-tolerance environment.^15^ Bregs have been implicated in the formation of tertiary lymphoid structures in certain tumors, in which they maintain an immunosuppressive state and protect the tumor from immune responses.^16–18^ For example, Bruno et al^19^ have shown that antigen-presenting, exhausted tumor-infiltrating B cells (CD19^+^CD20^+^CD69^+^CD27^−^CD21^−^) induce Tregs in vitro. Additionally, intrathymic B cells have been shown to contribute to the development and proliferation of natural Tregs^20–23^ and some Bregs secrete interleukin-10, which inhibits M1 macrophages, natural killer cells, and Th1 cells,^24^ contributing to a tolerogenic environment.^25^

To explore the roles of B cells in tolerance development, here we utilize single-cell RNA sequencing (scRNA-seq) and trajectory analysis of transplanted mouse allografts recovered at serial time points over a 6-month period. More specifically, we utilize this approach to characterize B cell population subtypes and gene expression signatures in tolerance development. For example, comparison of scRNA-seq data of accepted and rejected kidney allografts at 7 days posttransplant shows a much smaller cluster of infiltrating B cells in the rejecting grafts. Collectively, these observations extend previous reports in human studies that acceptance of renal transplants is associated with a B cell signature.^26–28^ While previous human studies have examined peripheral blood mononuclear cells, our analysis complements this work by investigating intragraft immune cells, thereby providing insight into the tolerance processes occurring in transplanted organs.

## 2. Methods

### 2.1. Mice

The C57BL/6J (B6, H2b) and DBA/2J (DBA, H2d) strains were purchased from Jackson Laboratories (Strain #: 000664, 000671). All mice were maintained under pathogen-free conditions in filter-top cages throughout the experiments with an automatic water system and were cared for according to methods approved by the American Association for the Accreditation of Laboratory Animal Care.

### 2.2. Kidney transplantation

Kidney transplantation was performed as detailed in a previous report.^29^ In brief, kidney allografts were procured together with the cuff of donor aorta and inferior vena cava. Vascular anastomoses were performed in an end-to-side manner. The ureter was anastomosed to the recipient’s urinary bladder. Bilateral recipient nephrectomy was performed in the same setting. The utilized acceptance model was DBA/2J kidney to C57BL/6J; the rejection model was C57BL/6J kidney to DBA/2J (Median survival time (MST) = 9–10 days).^12^ Depletion of Foxp3^+^ Treg by the systemic administration of diphtheria toxin (DT) using B6 Foxp3^DTR^ recipients was carried out as previously described.^11^

### 2.3. Isolation of renal and spleen cells

Prior to tissue collection, the kidney was perfused using a collagenase solution (1× Hanks’ balanced salt solution, 1 mL of collagenase A, Roche product #: 10103578001, and 3 μL of DNase I, Sigma-Aldrich, product #: 10104159001). The renal allograft was procured, manually minced, and further digested in a collagenase solution for 30 minutes. Any remaining undigested tissue was manually passed through a 70-μm strainer, washed 3 times, and resuspended using fluorescence-activated cell sorting (FACS) buffer (10× phosphate buffered saline, double distilled water, 2% fetal bovine serum, and 0.1% sodium azide). The recipient, or naïve spleen, was collected in RPMI (Corning, product #: 10–040-CV) and then minced using the plunger of a syringe and a 70-μm strainer. To remove red blood cells from spleen, 1 mL of ACK lysis buffer (Gibco, product #: A10492–01) was added. Cells were centrifuged and washed 3 times with phosphate buffered saline, followed by resuspension in FACS buffer.

### 2.4. Flow cytometry analysis

Kidney and spleen cells were initially stained with a viability dye (antigen-presenting cell [APC]-Cy7, 1:1000 dilution, Invitrogen, product #: L34976 A). Subsequently, CD16/32 Fc Block (1:100 dilution, BioLegend, product #: 101302) was added to each sample for a 5- to 10-minute preincubation step. After Fc Block, CD1d (After Fc Block, CD1d (fluorescein isothiocyanate, FITC), Tim1 (Phycoerythrin, PE), CD5 (Percp-Cy5.5), CD19 (APC), and CD45 (BV510) surface markers were added to the samples in a 1:200 dilution for each antibody for 30 minutes (Supplementary File 1). Surface markers for immunoglobulin (Ig) A (PE), IgD (APC), and IgM (APC) were also stained using optimized dilutions for 30 minutes (Supplementary File 1). Following incubation of the surface markers, the samples were resuspended in FACS buffer and analyzed via flow cytometry on the BD FACsverse instrument. Gating strategies were controlled for using fluorescent minus-one controls and universal negatives (Supplementary Fig. S1).

### 2.5. Statistics

Two-sample t-tests were performed to assess the significance of population differences regarding mean fluorescence intensities with flow cytometry markers (eg, CD19).

Gene expression statistics were carried out for various cell clusters and time points based on the Seurat statistical package (FindMarkers).^30^ This method used the Wilcoxon rank-sum test (default), the student’s t-test, logistics regression, and the DESeq2 method (based on negative binomial distribution).^31^ The 2 main statistical tests from this package used within this work were the Wilcoxon rank-sum and the DESeq2 method.

## 3. Results

### 3.1. Single-cell RNA sequencing analysis reveals a temporal shift from a T cell–rich immune microenvironment to a B cell–rich immune microenvironment within accepted kidney allografts

**Figure 1.**
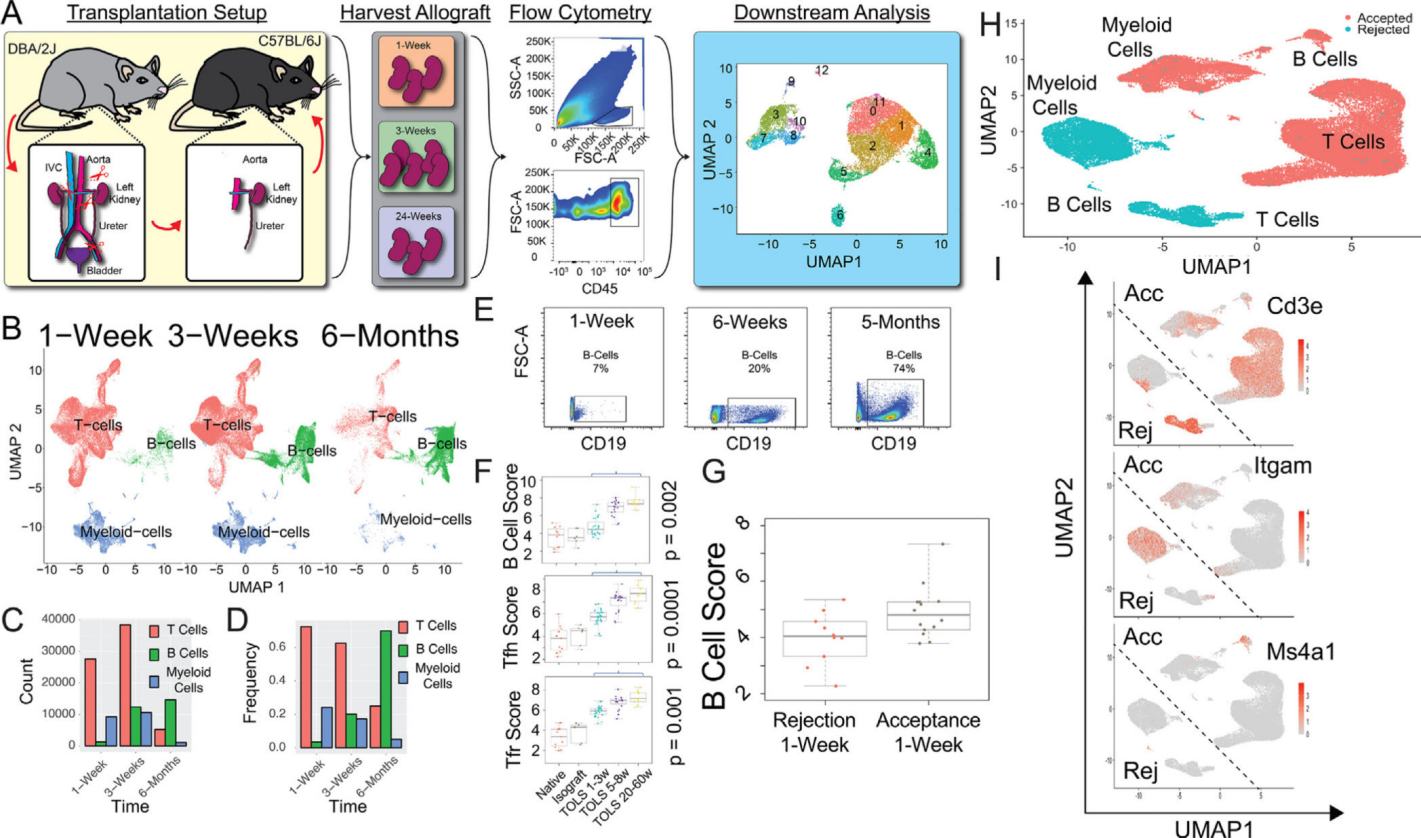
Temporal single-cell RNA sequencing cluster analysis of immune cells isolated from accepted kidney allografts. (A) Schematic illustration of the experimental setup. (B) UMAP represents 3 main clusters (T cells, B cells, and myeloid-derived cells) found in DBA/2 kidneys transplanted into B6 mice at 3 different time points. scRNA-seq data are integrated and time-separated (1-week, N = 3 mice; 3-week, N = 5 mice; and 6-month, N = 3 mice). Each dot represents a cell, and each group of colored cells represents a different major cell type cluster. The Seurat statistical package for FindMarkers (DESeq2 method) showed statistically significant gene expression differences between the 3 main cluster types (T cells, B cells, and myeloid cells). (C) Raw cell number data of cells specifically expressing T cell, B cell, and myeloid cell-specific genes over the 3 time points from UMAP in panel B. (D) Frequencies of each main cell type within the CD45+ population over the 3 time points from UMAP in panel B. (E) Flow cytometric analysis of CD19+ cells within the total cell population isolated from accepted kidney allografts at 1 week, 6 weeks, and 5 months posttransplant. A 2-sample t-test was performed on the mean fluorescence intensity between 1-week and 5-months and had a *P* value of 2.45 × 10^−8^. (F) Bulk RNA analysis of tissue sections of accepted kidney allografts from 1-week to 60-weeks posttransplant shows a progressive increase in the B, T follicular (Tfh), and T follicular regulatory (Tfr) cell scores (*P* = .002, *P* = .0001, and *P* = .001, respectively). (G) Bulk RNA analysis of tissue sections shows a greater B cell signature in accepted allografts compared with rejecting kidney allografts. (H) UMAP represents the integrated scRNA-seq data of accepted (n = 3) and rejecting (n = 2) kidney allografts, showing 3 distinct cellular clusters of CD45^+^ cells−T cells, B cells, and myeloid cells. (I) Feature map analysis shows expression analysis of T cells (*Cd3e),* myeloid cells *(Itgam)*, and B cells (*Ms4a1*). Acc, accepted; DBA/2J, Dilute Brown Agouti mouse strain; FSC, Forward Scatter; Rej, rejected; scRNA-seq, single-cell RNA sequencing; SSC, Side Scatter; TOLS, Treg-rich organized lymphoid structures; UMAP, Uniform Manifold Approximation and Projection.

We have previously reported that accepted kidney allografts form TOLS, which represent rTLOs.^10^ Bulk RNA sequencing and immunohistochemical analyses have shown that these structures consist of various immune cell types, including CD4^+^ and CD8^+^ T cells, myeloid cells, and B cells.^10^ To further elucidate the cellular makeup of the TOLS, we performed scRNA-seq analysis of purified CD45^+^ cells from accepted kidney allografts at 1-week, 3-week, and 6-month posttransplant (Fig. 1A). Seurat software packages^32^ for scRNA-seq data identified 3 major cell populations across timepoints: T cells, B cells, and myeloid cells (Fig. 1B). Forty to fifty percent of transcripts at 1 to 3 weeks for transplantation are associated with a CD8^+^ T cell phenotype that includes an exhausted signature (ie, *Pcdc1*, *Lag3*, and *Tox*). Interestingly, the macrophage population within the myeloid cluster had an M1 phenotype (ie, S100a8, Il6, Trem3, Ifng, Lrg1, Flt1, Tnfaiip3, Ccr7, and Traf1) at 1 week, and by 3 weeks, an increase of M2 specific transcripts (ie, ApoE, Trf, Selenop, and Chil3) within the M1 cluster was observed. These results may suggest a reprogramming of M1, inflammatory macrophages, to the more regulatory M2 phenotype.^33^ Strikingly, we found that over time, T cell and myeloid populations decreased across the 6-month posttransplant period, while B cells continued to increase during this time span (Fig. 1B–D). The absolute cell number within the allografts (ie, count; Fig. 1C) showed that the majority of CD45^+^ cells at 6-month expressed B cell-specific genes when compared with the number of myeloid and T cells and that B cells represented about 70% of the total CD45^+^ cells (Fig. 1D).

Flow cytometric analysis of total, viable CD45^+^ single cells isolated from kidney allografts demonstrated a statistically significant increase of CD19^+^ B cells from 1 week to 5 months (7% at 1 week, 20% at 3 weeks, and 74% at 5 months, respectively; Fig. 1E and Supplementary Fig. S1), which validated the scRNA-seq data (Fig. 1D). This is in contrast to T cell and myeloid cell populations, which show a temporal decline in frequency (ie, 72% at 1 week, 63% at 3 weeks, and 25% at 6 months for T cells, and 24% at 1 week, 17% at 3 weeks, and 5% at 6 months for myeloid cells; Fig. 1B–D). In addition, bulk RNA analysis of accepted kidney tissue samples from 1 to 60 weeks shows a progressive increase in the B cell score (*P* =.002), corroborating the scRNA-seq data (Fig. 1F). Interestingly, the T follicular (*Bcl6*, *Cxcr5*, *Cxcl13*; *P* = .0001) and the T follicular regulatory (*Bcl6*, *Cxcr5*, *Cxcl13*, *Foxp3*; *P* = .001) cell scores parallel the B cell data, showing a progressive increase with time (Fig. 1F). This contrasts with the reductions we see in the CD8^+^ and CD4^+^ T cell populations over time.

Lastly, bulk mRNA analysis using NanoString nCounter of rejected and accepted grafts shows higher levels of B cell transcripts in accepted grafts when compared with rejecting grafts at 1 week after transplant (*P* = .025) (Fig.1G). The corresponding scRNA-seq data analysis of CD45^+^ cells isolated from rejecting kidney allografts undergoing T cell-mediated rejection (n = 2) reveals B cell numbers are also reduced in a rejecting kidney model when compared with an accepted kidney at day 7 posttransplant (Fig. 1H, I). Interestingly, analysis of the integrated data of rejecting vs accepting kidney allografts shows that there are distinct T and B cell clusters as well as distinct myeloid cell clusters that define rejection vs acceptance (Fig. 1H, I).

### 3.2. Temporal cluster analysis of B cell subsets within the accepted renal allografts

**Figure 2.**
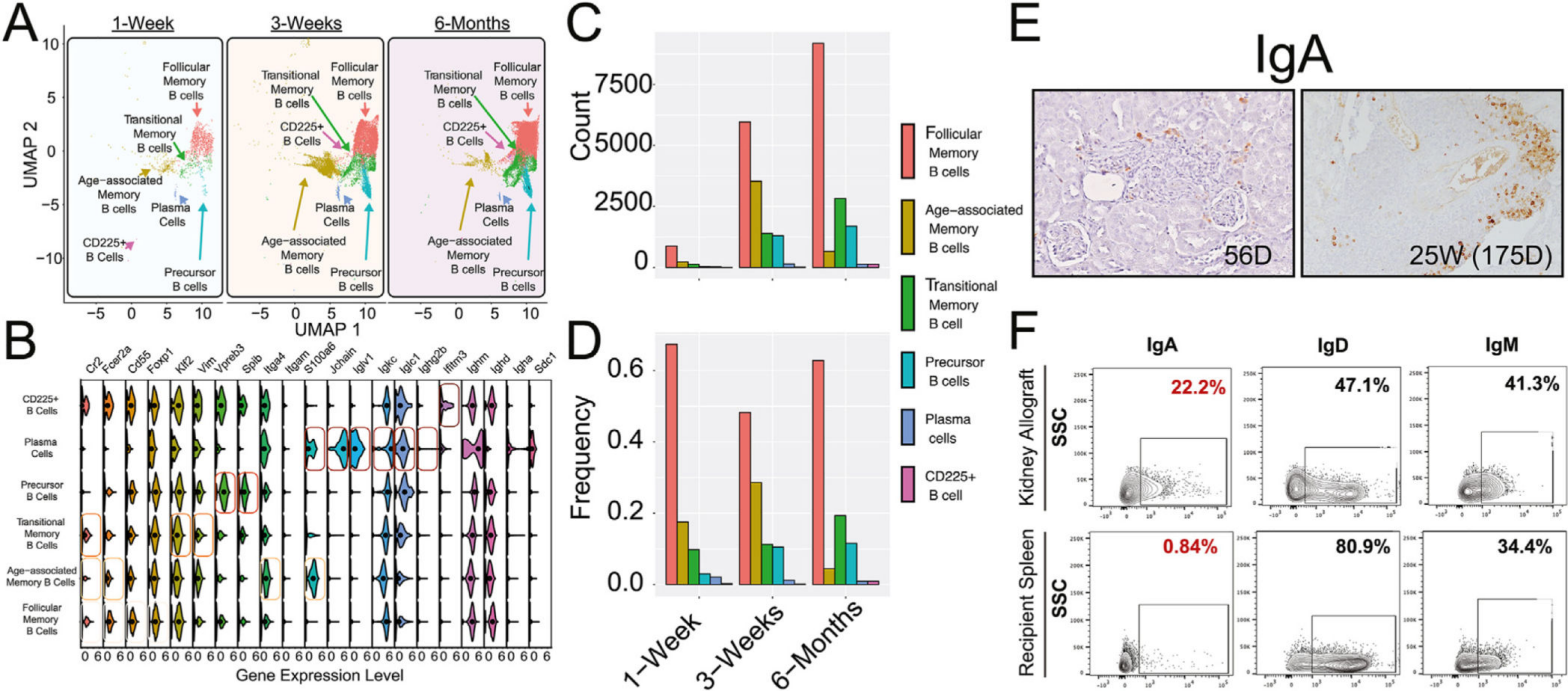
Single-cell RNA sequencing time course and gene expression analysis within B cell clusters. (A) Subpopulation analysis of B cell clusters broken down by time points. There were 6 B cell populations that emerged at different frequencies at the 1-week, 3-week, and 6-month time points. Each dot represents a cell, and each group of colored cells represents a different cell type cluster. (B) Violin plot showing gene expression analysis for canonical markers for the 6 B cell populations observed. Each row represents a particular B cell cluster (eg, plasma cells), whereas each column represents a specific gene (eg, *Cr2*). Violin plots represent the distributions of gene expression across biological replicates (1-week, n = 3; 3-week, n = 5; 6-month, n = 3). Black circles represent median gene expression value of distributions. The Seurat statistical package for FindMarkers (DESeq2 method) showed statistically significant gene expression differences between all 6 subtypes of B cells. (C) Count of each cell cluster over the 3 time points from the data in panel A. (D) Frequencies of each B cell cluster as a percentage of total B cell populations over the 3 time points. (E) IgA staining within rTLOs in DBA kidneys 56 and 175 days after transplantation. Original magnification, 40× for 56-day tissue sample and 20× for 175-day tissue sample. Each image is representative of 3 different tissue samples. (F) Flow cytometric analysis of IgA, IgD, and IgM expression on gated B cells at 25 weeks posttransplant. The data are representative of 3 different experiments. DBA/2J: Dilute Brown Agouti mouse strain 2J; Ig, immunoglobulin; rTLO, regulatory tertiary lymphoid organs.

We next investigated the observed B cell cluster by using markers for various B cell subtypes (Supplementary Table S1)^34–44^ and identified multiple B cell populations that included follicular memory *(Cr2*^*int*^*, Fcer2a*^*hi*^*, Cd55*^*hi*^, and *Foxp1*^*hi*^), transitional memory (*Cr2*^l◦^, *Fcer2a*^int^, *Klf2*^hi^, and *Vim*^hi^), precursor (*Vpreb3*^hi^ and *Spib*^hi^), age-associated memory (*Cr2*^l◦^, *Fcer2a*^l◦^, *Itga4*^hi^, and *S100a6*^int^), CD225^+^ (*Ifitm3*^hi^) B cells, and plasma cells (*Jchain*^hi^, *Iglv1*^hi^, *Igkc*^hi^, *Iglc*1^hi^, *Ighm*^hi^*, Igha*^hi^, and *Sdc1*^hi^) (Fig. 2A, B). As shown in Figure 1, the B cell subtypes demonstrated changing temporal profiles over the 6-month experimental period within the allografts. These canonical markers revealed unique gene expression patterns for each of the 6 B cell subtypes. Many of the canonical markers showed expression in multiple cell types, but certain markers such as Jchain or S100a6 demonstrated high specificity within certain B cell subtypes (ie, plasma cells and age-associated memory B cells, respectively). While these B cell subsets all increased in cell number between 1-week and 3-week (Fig. 2C), the follicular memory and transitional B cell subsets represented the 2 major populations within the B cell cluster by 6 months (Fig. 2C, D). Age-associated memory B cells had an initial rise in cell number and allograft cellular frequency at 3 weeks but decreased by 6 months. Precursor B cells, on the other hand, increased in cell number and frequency across all time points. Lastly, plasma cells and CD225^+^ B cells remained a small percentage of B cells within the allografts across all time points.

Analysis of these cell types for antibodies revealed that *Ighm* and *Ighd* are the predominantly expressed Ig genes at 1, 3, and 24 weeks, with very little or no expression of *Ighg2b* (Fig. 2D). Additionally, immunohistochemical staining for IgA shows an increase of IgA-expressing cells within the rTLOs in accepted kidney allografts at 24-week compared with 8-week samples (Fig. 2E). These results were reconfirmed via flow cytometric analysis, which showed that IgM, IgD, and IgA are expressed on B cells at 24 weeks (Fig. 2F).

**Figure 3.**
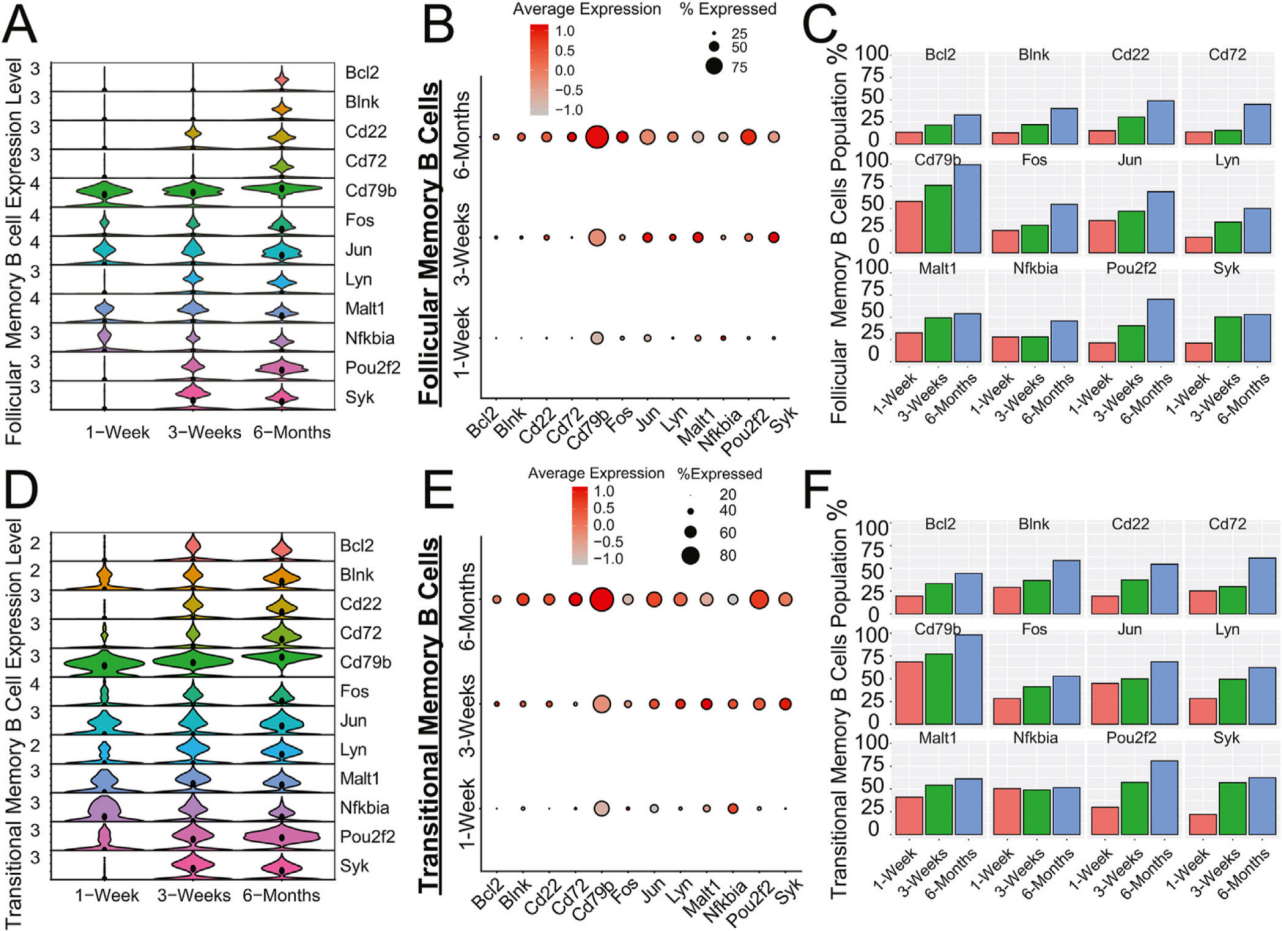
Single-cell RNA sequencing analysis of the B cell receptor (BCR) signaling cascade. (A) Violin plot of 12 genes (of 63 [Supplementary Table 2]) involved in the BCR signaling cascade within the follicular memory B cell population. Violin plots are depicted over 3 time points, including at 1-week, 3-week, and 6-month posttransplant. Black circles represent median gene expression value of distributions. The Seurat statistical package for FindMarkers (Wilcoxon rank-sum test) showed statistically significant gene expression differences between 1 and 3 weeks for *Syk*, *Pou2f2*, *Lyn*, *Cd79b*, *Cd22*, *Malt1*, *Blnk*, *Jun*, and *Bcl2*, with *P* values ranging from 7.96 × 10^−7^ to 2.6 × 10^−51^. The Seurat statistical package showed statistically significant gene expression differences between 1-week to 6-month for *CD79b*, *Pou2f2*, *Cd22*, *Cd72*, *Lyn*, *Fos*, *Malt1*, and *Nfkbia*, with *P* values ranging from 3.6 × 10^−6^ to 1.6 × 10^−201^. (B) Dot plot representation of the follicular memory B cells with the same genes and time points in subpanel A. The larger the dot in the graph, the higher the percentage of the population expressing the gene. The redder, the higher the gene expression, and the grayer, the lower the gene expression. (C) Frequency graphs of the same 12 genes as subpanels A and B for follicular memory B cells across the 3 time points. Frequency represents the percentage of the population that expresses the gene in question. (D) Violin plot of the same 12 genes (out of 63) involved in the BCR signaling cascade within the transitional memory B cell population. Violin plots are depicted over 3 time points, including at 1-week, 3-week, and 6-month posttransplant. Black circles represent median gene expression value of distributions. The Seurat statistical package (FindMarkers) showed statistically significant gene expression differences between 1 and 3 weeks for *Syk, Pou2f2, Lyn, Cd79b, Cd22, Bcl2, Malt1*, and *Fos*, with *P* values ranging from 0.01 to 3.07 × 10^−15^. The Seurat statistical package (*FindMarkers)* showed statistically significant gene expression differences between 1 week and 6 months for *Cd79b, Pou2f2, Syk, Cd22, Lyn, Cd72, Blnk, Bcl2, Fos, Jun*, and *Nfkbia*, with *P* values ranging from 0.036 to 6.6 × 10^−45^. (E) Dot plot representation of the transitional memory B cells with the same genes and time points in subpanel D. The larger the dot in the graph, the higher the percentage of the population expressing the gene. The redder, the higher the gene expression, and the grayer, the lower the gene expression. (F) Frequency graphs of the same 12 genes as subpanels D and E for transitional memory B cells across the 3 time points. Frequency represents what percentage of the population expresses the gene in question.

Given the shift from a T cell– to a B cell–rich immune microenvironment in accepted kidney allografts along with the identification of several B cell subsets, we next analyzed the temporal expression of genes associated with B cell receptor (BCR) signaling. We investigated 63 genes involved in the BCR signaling cascade^45,46^ (Supplementary Table S2). Analysis of BCR genes within the 2 largest B cell subset populations (ie, follicular and transitional memory B cells) revealed BCR signaling cascade genes were elevated over time posttransplant, paralleling the shift from a T cell– to a B cell–rich environment (Fig. 3). A subset of the strongest dose-responsive changes of the 63 BCR signaling cascade genes were plotted, including *Bcl2*, *Blnk*, *Cd22*, *Cd72*, *Cd79b*, *Fos*, *Jun*, *Lyn*, *Malt1*, *Nfkbia*, *Pou2f2*, and *Syk* (Fig. 3A, D), with lower expression of these genes observed in the rejecting kidney grafts (Supplementary Fig. S2A). For both follicular memory and transitional memory B cells, BCR-associated genes showed low basal expression at 1 week and a marked increase in expression at 3 weeks posttransplant, which was maintained or further increased at the 6-month time point (Fig. 3A, D). This increased pattern of gene expression was also observed with follicular memory and transitional memory B cells (Fig. 3B, C, E, and F). Interestingly, we observed the expression of *Ighv1-*55 in the 3 6-month samples, which may suggest BCR clonality, but this will require further future analysis.

### 3.3. Trajectory analysis reveals multistate intragraft differentiation of B cells

**Figure 4.**
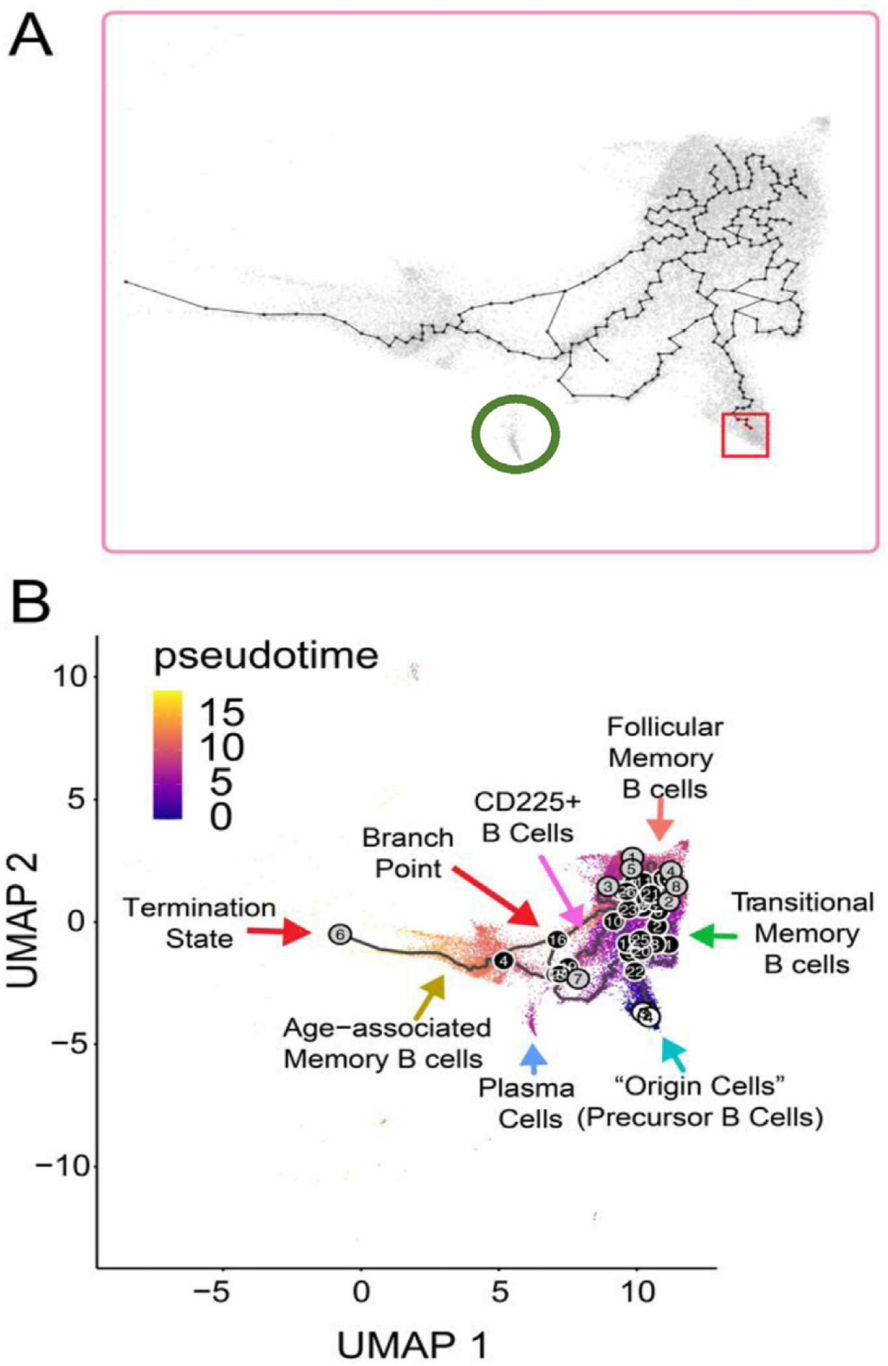
Single-cell RNA sequencing trajectory analysis of B cells. (A) Monocle 3 map illustrating trajectory nodes and origin cells (precursor B cells) for the B cell data set. The red square highlights nodes within Monocle data set chosen as the origin cells, which are represented by the precursor B cell population. The green circle highlights the plasma cell population. (B) Monocle trajectory analysis for B cell populations. White circles represent starting origin points (precursor B cells). Black/gray lines represent trajectories taken for cell types. Gray circles represent termination states of cell trajectories. Black circles represent branch points that occur in the B cell trajectories taken. Pseudotime is overlaid on the UMAP from Figure 3 that is on a gradient color scale (purple to yellow representing less time to longer time needed to reach a given state, respectively). UMAP, Uniform Manifold Approximation and Projection.

The temporal shift from a T cell– to a B cell–rich signature raised the question of whether there were cellular transitions occurring within the transplanted allograft. To address whether there was intragraft B cell differentiation or development, we performed trajectory analysis for the B cell subtypes using Monocle 3 on the scRNA-seq data^47,48^ for the B cell subtypes (Fig. 4A). The Monocle 3 software platform allows the determination of termination cellular states, intermediate states, and potential starting states within the transplanted allografts. To determine an accurate trajectory analysis of the B cells within the transplanted allograft, “cells of origin” were selected in Monocle 3^47,49–51^ (Fig. 4A, red box). For B cell trajectories, we selected the precursor B cell cluster as the point of origin since these cells have the potential of differentiating into various B cell subtypes.^52^

Analysis of our integrated scRNA-seq data using precursor B cells as the origin state resulted in a trajectory analysis of multiple paths and termination points (Fig. 4B). More specifically, trajectory analysis revealed that precursor B cells developed into follicular memory B cells, transitional memory B cells, and age-associated memory B cells within the accepted kidney graft (Fig. 4A, B). However, precursor B cells did not differentiate into plasma cells or CD225^+^ B cells (Fig. 4A, B), which indicates that these cells likely entered the transplanted kidney in a differentiated state. The lack of terminal differentiation nodes (Fig. 4B, gray circles) can be seen in CD225^+^ B cells and plasma cells, which is based on gene expression profiles of the B cell subtypes. In addition, pseudotime can be used as a metric to investigate the speed with which cell types transition to other states. In the case of the B cell subtypes, pseudotime overlaying the Uniform Manifold Approximation and Projection (UMAP) showed that differentiation to transitional and follicular memory B cells occurs before differentiation to age-associated memory B cells (Fig. 4B) and transitional B cells exhibited the shortest pseudotime for differentiation.

### 3.4. A temporal analysis reveals the intragraft development of a regulatory B cell signature

**Figure 5.**
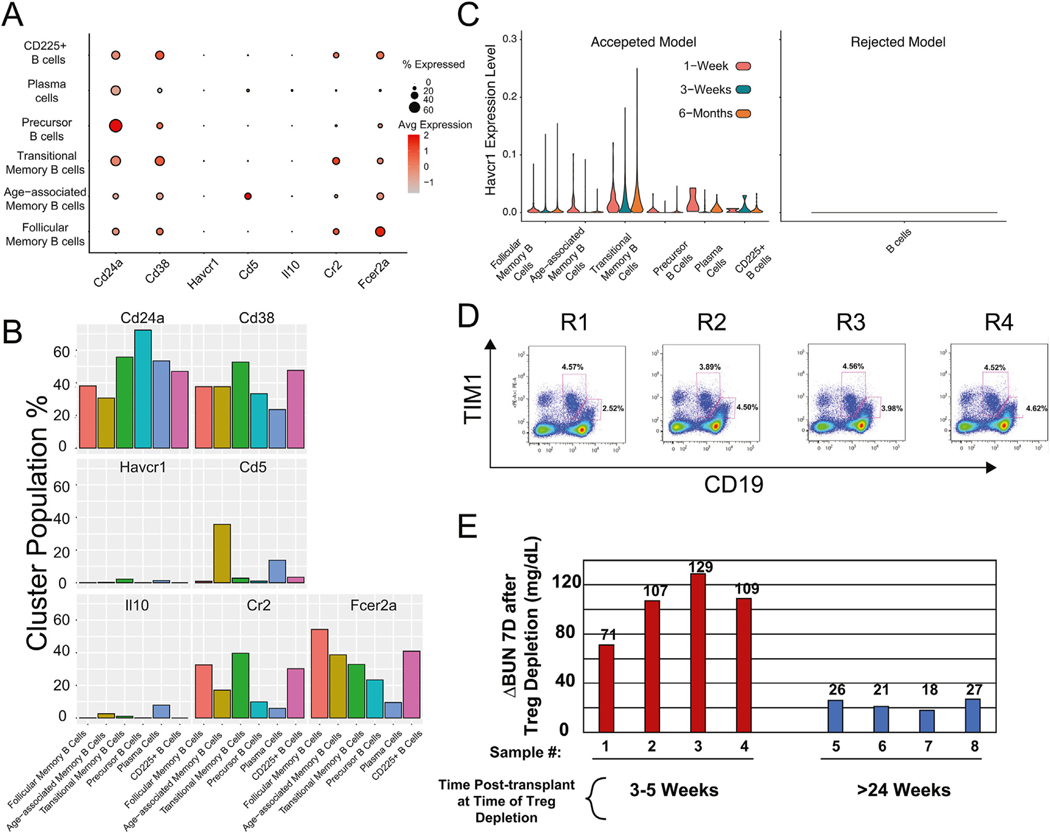
Regulatory B cell signature. (A) Dot plot showing gene expression levels of *Cd5*, *Cd24a*, *Cd38*, *Cr2*, *Fcer2a*, *Il10*, and *Havcr1* genes related to Breg phenotypes. The size of the dot refers to the percentage of cells that express the gene within the individual B cell subset. The redder, the higher the gene expression, and the grayer, the lower the gene expression. (B) Population percentage of 6 B cell clusters expressing *Cd5*, *Cd24a*, *Cd38*, *Cr2*, *Fcer2a*, *Il10*, and *Havcr1*. (C) Violin plot gene expression levels of *Havcr1* (TIM-1) within the various B cell clusters at 1 week, 3 weeks, and 6 months posttransplant in accepted kidney allografts vs *Havcr1* expression in the B cell cluster in rejecting kidney allografts at 1-week posttransplant. (D) Flow cytometric analysis of CD45^+^ sorted B cells stained for CD19 and TIM-1 at 20 weeks shows the percentages of CD19^+^hiTIM-1^+^ and CD19+dimTIM-1^+^ cells. The graphs shown are representative cells isolated from accepted kidney allografts from 4 different recipients. (E) DBA/2 kidneys were transplanted into C57BL/6J.Foxp3^DTR^ recipients. Specific depletion of Foxp3^+^ regulatory T cells was carried out by the systemic administration of DT at early (3–5 weeks) and late (>24 weeks) stages posttransplant, as shown by the difference in circulating blood urea nitrogen levels between pre-DT treatment and 7 days post-DT treatment. Breg, regulatory B cell; DT, diphtheria toxin; TIM-1, T cell immunoglobulin domain and mucin domain-1; Treg, regulatory T cell.

Further temporal analysis of scRNA-seq data suggested that regulatory-like B cells^15,53,54^ are present in accepted kidney allografts based on increased expression of Breg markers within the B cell cluster, including *Cd5*, *Cd24a*, *Cd38*, *Cr2*, *Fcer2a*, *Il10*, and *Havcr1* (Fig. 5A, B). For interleukin-10 and T cell immunoglobulin domain and mucin domain-1 (TIM-1), there was a detectable population of cells expressing these genes (Fig. 5A, B). Furthermore, scRNA-seq analysis demonstrated *Havcr1* (TIM-1) expression within accepted kidney allografts across multiple time points posttransplant (Fig. 5C). For several B cell populations, such as follicular memory and transitional memory B cells, expression of *Havcr1* was often highest at 6 months posttransplant. In contrast, we observed essentially no *Havcr1* gene expression in CD45^+^ cells isolated from rejecting kidney allografts (Fig. 5C). Similar analysis was performed for other Breg markers (Supplementary Fig. S2B), which again showed expression in the accepted kidneys but minimal expression in the rejection model.

To complement the above scRNA-seq data, flow cytometry was performed on immune cells from accepted kidney allografts at 20 weeks posttransplant, which revealed the presence of CD19+hiTIM-1^+^ and CD19+dimTIM-1^+^ cells (Fig. 5D), with a smaller percentage of CD19^+^CD5^+^CD1d^+^TIM-1^+^ B cells (B10/TIM-1 B cells,^15,53,54^ Supplementary Fig. S3). In addition to B10/TIM-1 B cells, we also identified the expression of markers that reflect T1 (CD24^+^IgM^+^CD93^+^), T2 (CD24^+^CD21^+^CD23^+^ IgM^+^IgD^+^CD93^+^), and T3 (CD24^+^CD21^+^CD23^+^IgM^+^IgD^+^ CD93^+^CD62L^+^) transitional B cell phenotypes as well as marginal zone B cells^55,56^ starting at 3 weeks posttransplant, suggesting additional diversity of regulatory cells within these grafts. Furthermore, depletion of Tregs at 3 to 5 weeks after transplant of DBA kidneys into B6 recipients resulted in graft rejection within 10 days with a dramatic increase in circulating blood urea nitrogen levels (Fig. 5E). By contrast, however, depletion of Tregs >24 weeks posttransplant resulted in very slow or no rejection of the kidney allografts (Fig. 5E). While these findings may suggest deletional tolerance, our data raises the possibility that there is a transition from a Treg to a Breg mode of regulation. This will require future analysis.

### 3.5. Differential Siglec-G gene expression increases over time across most B cell subset populations

**Figure 6.**
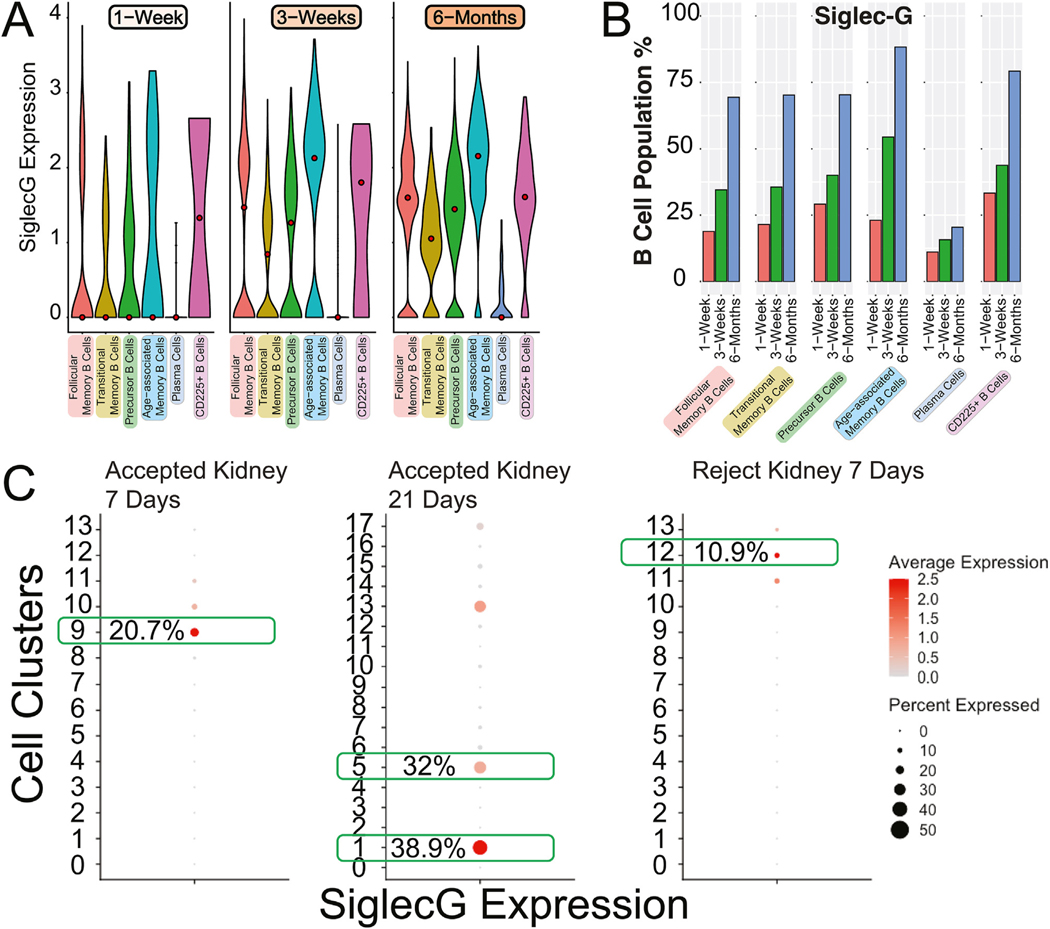
Siglec-G expression across B cell populations. (A) Violin plots of Siglec-G expression across 3 different time points (1 week, 3 weeks, and 6 months) posttransplant. Six B cell subsets shown include follicular memory, transitional memory, precursor, age-associated memory, and CD225^+^ B cells, as well as plasma cells. The Seurat statistical package (FindMarkers) showed Siglec-G gene expression to be statistically significantly different in follicular memory B cells from 1 to 3 weeks (*P*, 1.2 × 10^−18^) and from 1 week to 6 months (*P*, 1.3 × 10^−108^); to be statistically significantly different in age-associated memory B cells from 1 week to 3 weeks (*P*, 3.3 × 10^−5^) and from 1 week to 6 months (*P*, 4.8 × 10^−27^); to be statistically significantly different in transitional memory B cells from 1 week to 3 weeks (*P*, .00068) and from 1 week to 6 months (*P*, 5.3 × 10^−20^); to be statistically significantly different in precursor B cells from 1 week to 3 weeks (*P*, .00059) and from 1 week to 6 months (*P*, 2.0 × 10^−10^); to be statistically insignificant in plasma cells and CD225+ B cells from 1 to 3 weeks and from 1 week to 6 months. (B) Frequency graph of the same populations from panel (A) expressing Siglec-G at the same time points. (C) Dot plot analysis of single-cell RNA sequencing data of Siglec-G expression in accepted (1- and 3-week posttransplant) and rejecting (1-week posttransplant) samples. Boxed in green are the B cell clusters, and the number within each box refers to the percent of cells within that cluster that express Siglec-G.

We next explored genes that affect inflammation, regulation of proliferation, and influence over antibody production as potential features affecting tolerance development. One gene that emerged showing differential gene expression across populations and times was Siglec-G. This marker is known to be expressed on B cells, where it prevents the secretion of antibodies. It can also interact with CD22 on other cell types, where it can promote the upregulation of Siglec-G on their surface.^57,58^ Analysis of the Siglec-G gene revealed the highest expression in the CD225+ B cell population 1-week posttransplant (Fig. 6A, B). However, this gene was also expressed in follicular memory, transitional memory, precursor, and age-associated memory B cells to varying degrees posttransplant, with plasma cells exhibiting the lowest levels of expression.

Interestingly, by 6 months posttransplant, similar gene expression levels and population frequencies were found between the different B cell subtypes. Expression of Siglec-G was maintained across all subtypes at 6 months posttransplant, with the highest frequency of expression in age-associated memory B cells (Fig. 6A, B). Dot plot analysis revealed that *Siglec-G* is expressed with higher frequency in B cells from accepted compared to rejecting kidney allografts (Fig. 6C). Based on the regulatory function that has been associated with Siglec-G,^57,58^ this increased expression of Siglec-G in B cells from a tolerant kidney may point toward a mechanism that aids in long-term acceptance and tolerance formation.

## 4. Discussion

We have previously shown that in certain mouse strain combinations, kidney allografts are spontaneously accepted through the induction of natural tolerance.^10,11,13,29,59^ The main histopathologic characteristic of these accepted kidneys is the presence of TOLS, a novel rTLO that forms perivascularly and is made up of various immune cells.^10,11^ Here, we characterized immune cells in murine kidney grafts using scRNA-seq on CD45+ sorted cells isolated from accepted renal allografts at 1 week, 3 weeks, and 6 months and rejecting renal allografts at 1 week posttransplant. Analysis of scRNA-seq data revealed a temporal shift from a T cell–dominant profile to a B cell–rich immune environment by 6 months posttransplant in accepted allografts. This T cell–to–B cell shift was confirmed both as frequency and absolute cell number in the scRNA-seq data.

These results support the notion that B cells are increasing with time within the accepted kidney allografts, which is not solely due to reductions in the T cell population. Additionally, scRNA-seq analysis of infiltrating CD45^+^ cells in rejecting kidney allografts at 1-week posttransplant showed a vastly reduced B cell population when compared with accepted kidney allografts at a similar time posttransplant. Therefore, in this study, we focused on characterizing transcriptomic profiling at the single cell level of B cell populations within accepted kidney allografts.

In the acceptance transplant model presented here, we have not observed circulating IgM or IgG donor-specific antibodies.^11,13^ However, the single-cell investigation has identified follicular memory B cells and plasma cells within our accepted kidney allografts. To understand the role of these plasma cells, trajectory analysis was performed, which suggested that follicular memory B cells (or any other B cell type) do not differentiate into plasma cells within the graft. One potential feature relevant for these plasma cells may involve the *Sdc1* gene. *Sdc1* encodes for CD138, a marker for plasma cells,^60^ and we have previously shown that there are CD138^+^ cells within the TOLS structures of accepted kidney allografts.^10^ Our scRNA-seq data showed the presence of *Sdc1* within the plasma cell subcluster along with high *Ighm* (encodes for IgM) expression and negligible expression of *Ighg* (encodes for IgG). Further analysis of Igs using flow cytometry and immunohistochemistry showed that IgA is also expressed on B cells, especially at 6 months. While the implications of these findings need to be explored further, tumor-infiltrating IgA^+^ B cells, as opposed to IgG^+^ B cells, have been shown to contribute to a microenvironment that downregulates immune responses to tumors.^61^ It is possible that the presence of IgA^+^ cells in accepted kidney allografts serves a similarly protective function.

Additionally, the presence of nonsecreting *Sdc1*+*Ighm*+ plasma cells is not unprecedented, as previous studies have identified mature surface IgM-expressing plasma cells in mice and humans,^62,63^ where they can secrete interleukin-10 and possess regulatory function.^64–66^ In parallel to the potential importance of *Sdc1* for nonsecreting plasma cells, Siglec-G is a marker known to be expressed on B cells, with its main function involved in preventing the secretion of antibodies.^57,58^ Additionally, Siglec-G seems to be a B1 cell-specific inhibitory receptor, and Siglec-G-deficient mice exhibit a 10-fold increase in IgM serum levels.^67^ The work presented here has illustrated increased expression of Siglec-G within B cell subsets found in accepted kidney allografts. Taken together, these findings may suggest that plasma cells are being prevented from secreting antibodies locally; downregulation of antibody secretion may in turn prevent antibody-mediated rejection and promote tolerance of the kidney allograft.

In addition to diminished antibody secretion, Bregs have been shown to downregulate alloimmune responses^54,68^ and can also contribute to the inhibition of antitumor immune responses.^61,65,69^ B cell signatures have been associated with renal transplant tolerance in humans.^26–28^ Cherukuri et al^26^ showed that transitional B cell cytokines predict favorable renal allograft outcomes. While human studies have analyzed PMBC, the study here has aimed at characterizing intragraft B cell subsets. Although the functionality of B cell subsets identified here is still unknown, initial gene expression and flow cytometric analyses suggest the presence of a regulatory B cell signature. We hypothesize that this regulatory signature may be associated with long-term kidney allograft tolerance, with transitional memory B cells showing the greatest expression of *Havcr1*, the gene that encodes TIM-1. This observation potentially corroborates what has been previously observed in patients with accepted kidney allografts.^26–28^ Notably, while kidney allografts are rejected within a week following Treg depletion at 3 to 4 weeks posttransplant,^11^ we observed slower or no rejection following Treg depletion at 6 months posttransplant. These findings may point toward a shift from Treg- to Breg-mediated tolerance, wherein Bregs drive the tolerogenic environment within renal rTLOs at later stages. B cell depletion studies are currently underway at various timepoints posttransplant to assess kidney allograft survival.

In summary, scRNA-seq analysis of immune cells from accepted kidney allografts revealed a temporal shift from a T cell–to a B cell–rich immune microenvironment in long-term accepted kidney allografts. This is accompanied by an increased Breg signature within the B cell subsets, which mirrors observations in patients who have accepted kidney grafts.^26–28^ Future studies require additional investigations to understand the role(s) these B cell subsets play in the induction and/or maintenance of renal allograft tolerance and how they interact with other cells within transplanted allografts. Elucidating the significance of these cellular transitions within transplanted allografts may offer insights into tolerance development and opportunities to prevent rejection.

## Supplementary Material

Multimedia component 1

Multimedia component 2

Supplementary Figure 3

Supplementary Figure 4

Supplementary Figure 5

Supplementary Figure 2

Supplementary Figure 1

## Data Availability

The data generated and analyzed during this study are included within this article and its Supplementary Materials.

## Abbreviations:

Acc: accepted
BCR: B cell receptor
Breg: regulatory B cell
C57BL/6: Mouse Number 57’ or ‘C57 black 6 Mouse Strain’
DBA/2J: Dilute Brown Agouti mouse strain
DT: diphtheria toxin
FITC: fluorescein isothiocyanate
FSC: Forward Scatter
Ig: immunoglobulin
MST: Median Survival Time
PE: Phycoerythrin
Rej: rejected
scRNA-seq: single-cell RNA sequencing
rTLOs: regulatory tertiary lymphoid organs
SSC: Side Scatter
TIM-1: T cell immunoglobulin domain and mucin domain-1
Tfh: T follicular
Tfr: T follicular regulatory
TOLS: Treg-rich organized lymphoid structure
Treg: regulatory T cell
UMAP: Uniform Manifold Approximation and Projection
