# Supplementary material for: Intragraft B cell differentiation during the development of tolerance to kidney allografts is associated with a regulatory B cell signature revealed by single cell transcriptomics
Source: Am J Transplant. Author manuscript; Available in PMC 2024 Sep 1. (PMC11232115; doi:10.1016/j.ajt.2023.05.036)
Supplement: Multimedia component 2 [file NIHMS1998438-supplement-Multimedia_component_2.docx]

*S2.1. Single Cell RNA Sequencing*

Isolated kidney or spleen cells were sorted for viable CD45+ cells via flow cytometry using an anti-CD45+ antibody (BioLegend product number: 103114). These cells were used to construct single-cell RNA-seq libraries at Chromium 10x instrument using Chromium Next GEM Single Cell 3’ kit, which were sequenced in paired-end fashion^30^ on Illumina HiSeq 2500 instrument to the depth of approximately 100 million read pairs per sample.

*S2.2. Bioinformatics analyses of scRNA-seq data*

The raw sequencing data underwent initial mapping and processing using CellRanger package (Chromium Genomics). The resulting read counts were further analyzed using Seurat^31^ and Monocle^32-34^. This included filtering cells by the number of UMIs, mitochondrial content, and number of expressed genes, and further normalization and scaling of read counts. Datasets for various time points (1-week, 3-weeks, & 6-months) and biological replicates (Accepted Kidney: N = 3 at 1-week, N = 5 at 3-weeks, & N = 3 at 6-months; Rejecting Kidney: N = 2 at 1-week) were integrated, followed by principal component analysis (PCA), generation of UMAP plots, and cell clustering using Seurat functions with default parameters. Mouse RNA sample replicates were tagged individually and underwent scRNA-seq analysis separately. All mouse replicates were integrated together using Seurat after scRNA-seq was performed. Annotation of cell types was performed manually by investigating canonical markers in the literature. Analysis of gene expression across time and cluster type was performed using Seurat software packages. A total of 120,318 CD45+ cells from accepted kidneys were analyzed.

Integrated data from Seurat analysis was then transformed into a Monocle 3 object including expression data and cell-level metadata using the *as.cell_data_set* function to allow trajectory analysis (RStudio script). This object underwent further analysis including unsupervised clustering of cells using the *cluster_cells* function and to update the Monocle 3 object. Next, the Monocle 3 object underwent analysis using the *learn_graph* function to determine the biological program of gene expression for the scenario in question, and therefore learned the trajectory of cells through this higher-dimensional space. Lastly, the *order_cells* function was used to choose root states using the interactive Monocle 3 online software and determine the pseudotime values based on the object produced from using the *learn_graph* function. When this was complete, various qualitative graphs were produced to illustrate the trajectories of cell types in a larger population.

*S2.3. Histological and Immunopathological Analysis*

Sagittal sections of allografts were fixed in formalin. Immunohistochemistry was performed using IgA (RMA1, BioLegend, San Diego, CA). Whole slide scans were performed at 20x magnification (Aperio CS; Aperio, Vista, CA) and morphometric analysis was performed using Aperio Digital Pathology software (Leica Biosystems, Buffalo Grove, IL) image analysis algorithms. Brightfield microscopy was performed using an Olympus Bx53 microscope equipped with an Olympus DP27 camera.

*S2.4. NanoString Bulk RNA Analysis*

mRNA was extracted from formalin fixed paraffin embedded kidney allograft tissue samples from 1-week, 3-weeks, and 5-months post-transplantation as previously described^35,36^. Gene expression was assessed with a comprehensive Mouse Cancer Immunology probe set, which includes 770 genes related to the immune system, major cell pathways, and housekeeping genes. mRNA numbers were detected using the nCounter Max platform (NanoString Technologies, Seattle, WA). Normalized counts were analyzed with the Advanced Analysis software.
