# Supplementary material for: Intragraft B cell differentiation during the development of tolerance to kidney allografts is associated with a regulatory B cell signature revealed by single cell transcriptomics
Source: Am J Transplant. Author manuscript; Available in PMC 2024 Sep 1. (PMC11232115; doi:10.1016/j.ajt.2023.05.036)
Supplement: Supplementary Figure 1 [file NIHMS1998438-supplement-Supplementary_Figure_1.pptx]

## Slide 1
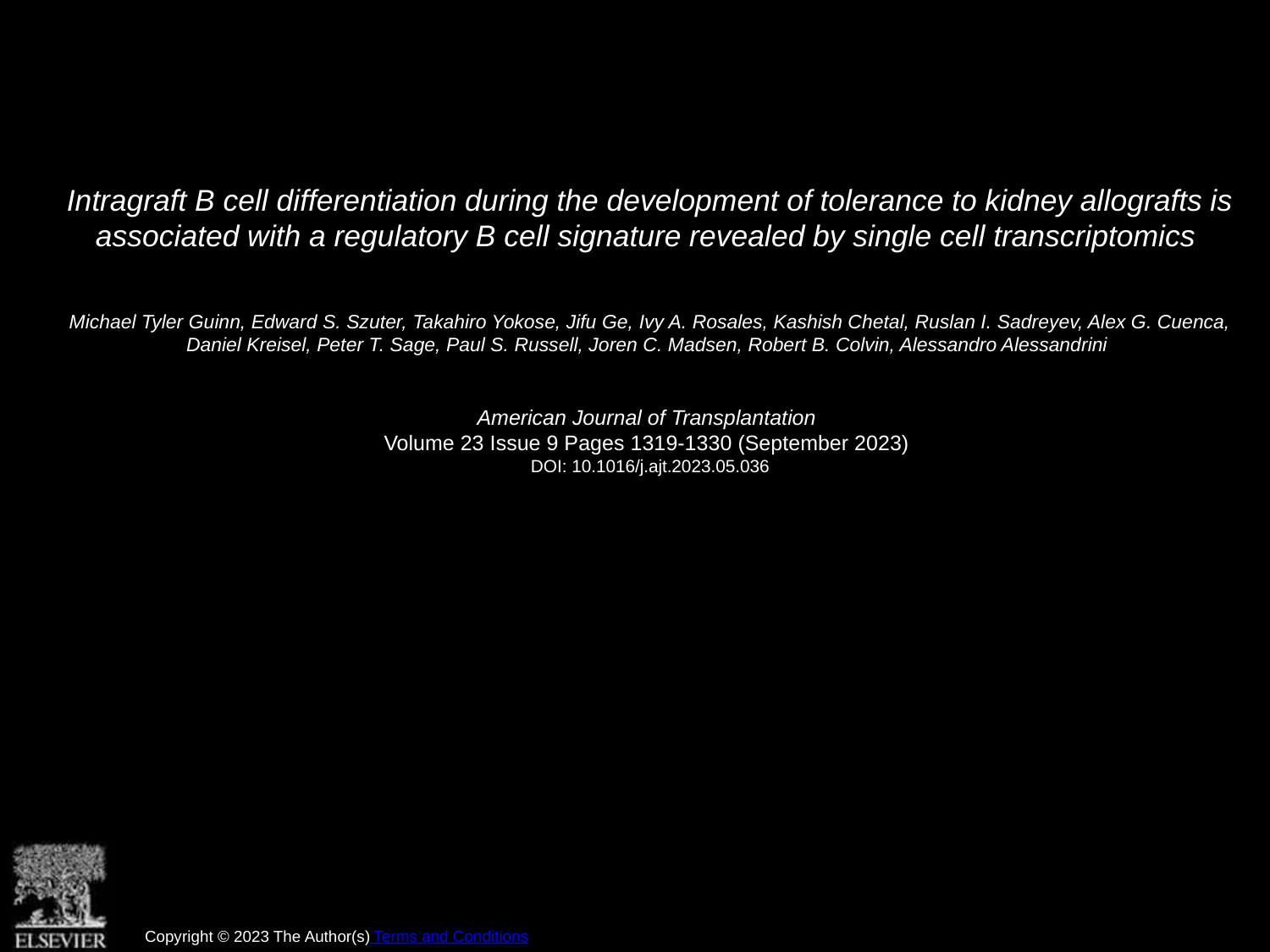

Intragraft B cell differentiation during the development of tolerance to kidney allografts is associated with a regulatory B cell signature revealed by single cell transcriptomics
Michael Tyler Guinn, Edward S. Szuter, Takahiro Yokose, Jifu Ge, Ivy A. Rosales, Kashish Chetal, Ruslan I. Sadreyev, Alex G. Cuenca, Daniel Kreisel, Peter T. Sage, Paul S. Russell, Joren C. Madsen, Robert B. Colvin, Alessandro Alessandrini
American Journal of Transplantation
Volume 23 Issue 9 Pages 1319-1330 (September 2023)
DOI: 10.1016/j.ajt.2023.05.036
Copyright © 2023 The Author(s) Terms and Conditions

## Slide 2
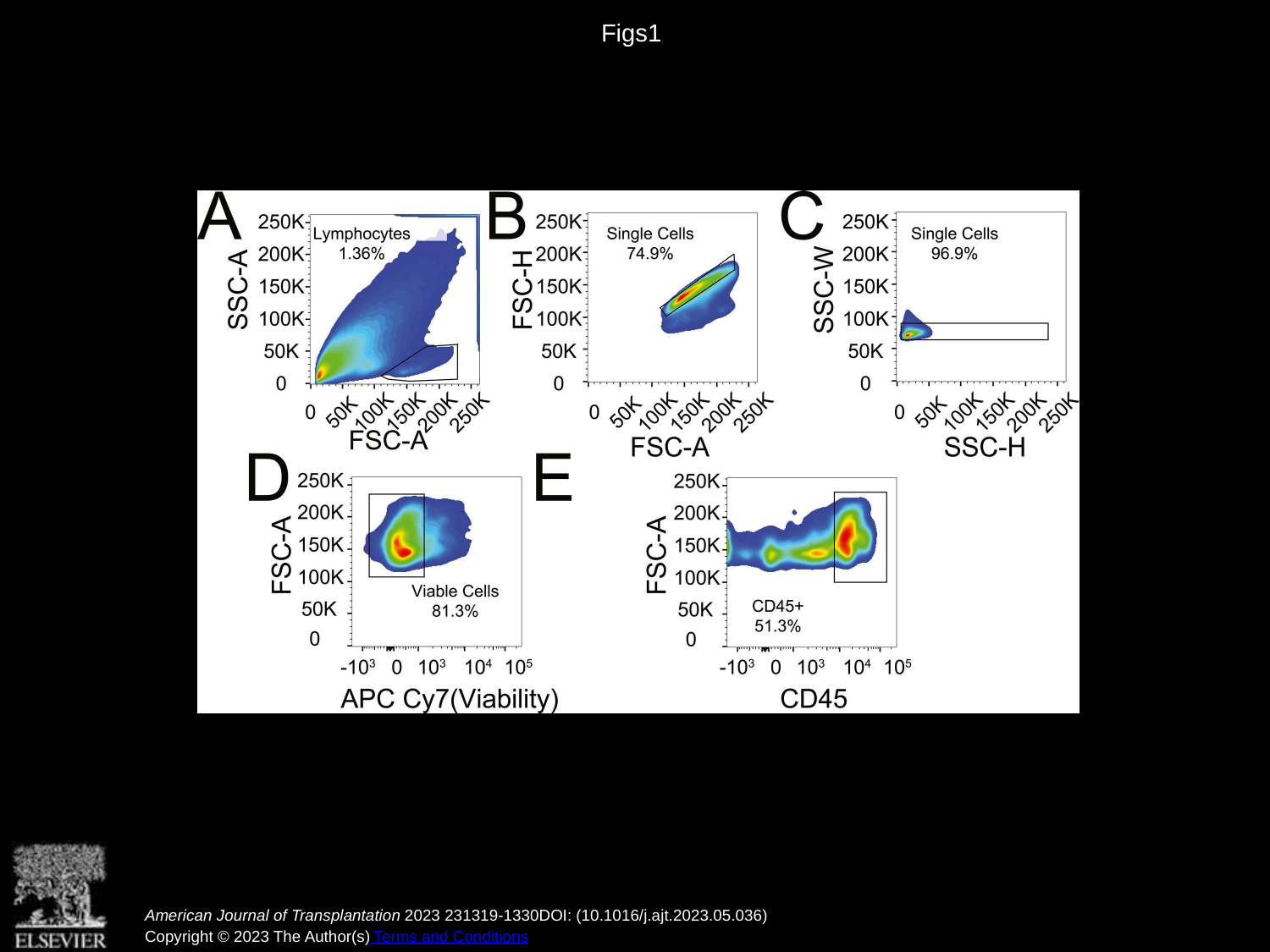

Figs1
American Journal of Transplantation 2023 231319-1330DOI: (10.1016/j.ajt.2023.05.036)
Copyright © 2023 The Author(s) Terms and Conditions
